# Supplementary material for: Splice-Junction-Based Mapping of Alternative Isoforms in the Human Proteome
Source: Cell Rep. Author manuscript; Available in PMC 2020 Jan 15. (PMC6961840; doi:10.1016/j.celrep.2019.11.026)
Supplement: 2 [file NIHMS1546469-supplement-2.zip › DF1/PXD010154/Thyroid/Thyroid_9_TG_NSDMGSRPESMGCR.pdf]

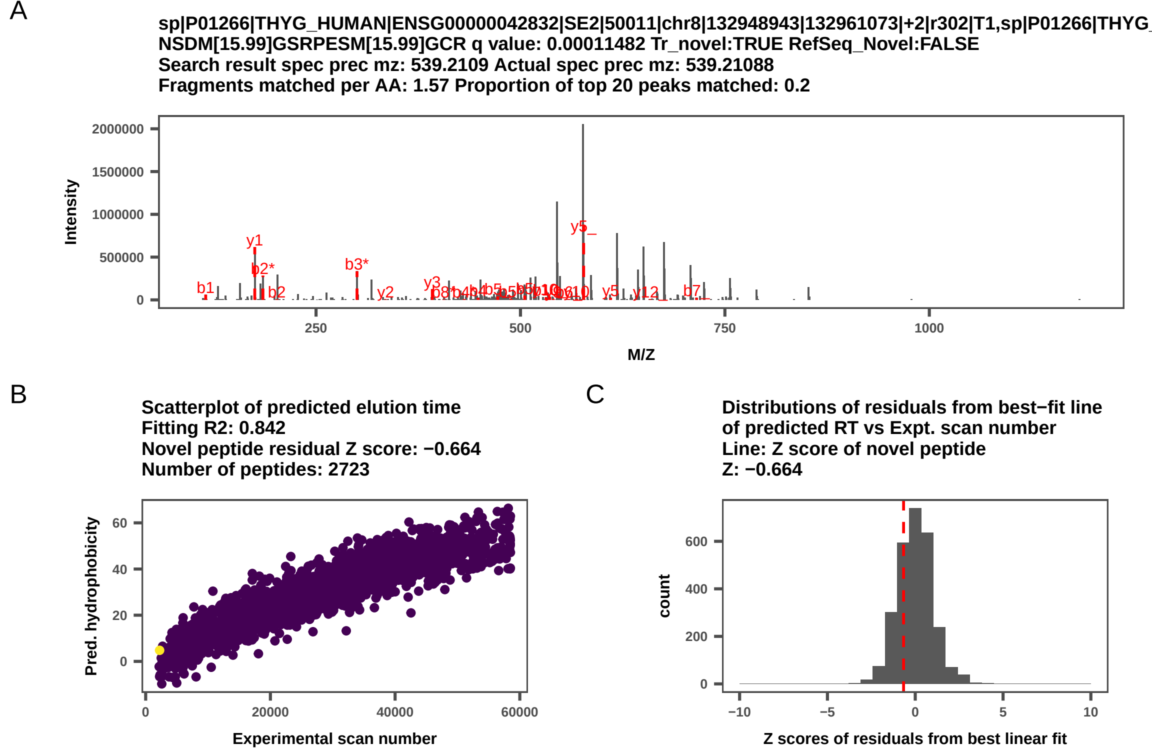
A
sp|P01266|THYG_HUMAN|ENSG00000042832|SE2|50011|chr8|132948943|132961073|+2|r302|T1,sp|P01266|THYG_
NSDM[15.99]GSRPESM[15.99]GCR q value: 0.00011482 Tr_novel:TRUE RefSeq_Novel:FALSE
Search result spec prec mz: 539.2109 Actual spec prec mz: 539.21088
Fragments matched per AA: 1.57 Proportion of top 20 peaks matched: 0.2
Intensity
2000000
1500000
1000000
500000
0
M/Z
250
500
750
1000
y1
b2*
b1
b2
b3*
y2
y3
y5_
y5
y12
b7
B
Scatterplot of predicted elution time
Fitting R2: 0.842
Novel peptide residual Z score: −0.664
Number of peptides: 2723
Pred. hydrophobicity
Experimental scan number
0
20000
40000
60000
60
40
20
0
C
Distributions of residuals from best−fit line
of predicted RT vs Expt. scan number
Line: Z score of novel peptide
Z: −0.664
count
600
400
200
0
−10
−5
0
5
10
Z scores of residuals from best linear fit
